# Using NS5B Sequencing for Hepatitis C Virus Genotyping Reveals Discordances with Commercial Platforms

**DOI:** 10.1371/journal.pone.0153754

**Published:** 2016-04-20

**Authors:** Natalia Chueca, Isidro Rivadulla, Rubén Lovatti, Gabriel Reina, Ana Blanco, Jose Angel Fernandez-Caballero, Laura Cardeñoso, Javier Rodriguez-Granjer, Miriam Fernandez-Alonso, Antonio Aguilera, Marta Alvarez, Juan Carlos Galán, Federico García

**Affiliations:** 1 Servicio de Microbiología, Complejo Hospitalario Universitario Granada—Hospital San Cecilio, Instituto de Investigación Biosanitaria IBS, Granada, Spain; 2 Departamento de Microbiología y Parasitología USC, Servicio de Microbiología Hospital Conxo-CHUS Santiago de Compostela, A Coruña, Spain; 3 Servicio de Microbiología, Hospital Ramón y Cajal, Instituto Ramón y Cajal de Investigación Sanitaria (IRYCIS), Madrid, Spain; CIBER en Epidemiología y Salud Pública (CIBERESP), Madrid, Spain; 4 Servicio de Microbiología, Clínica Universidad de Navarra, Navarra Institute for Health Research (IdiSNA), Pamplona, Spain; 5 Servicio de Microbiología, Hospital la Princesa, Madrid, Spain; 6 Servicio de Microbiología, Complejo Hospitalario Universitario Granada, Hospital Virgen de las Nieves, Granada, Spain; University of Cincinnati College of Medicine, UNITED STATES

## Abstract

We aimed to evaluate the correct assignment of HCV genotypes by three commercial methods—Trugene HCV genotyping kit (Siemens), VERSANT HCV Genotype 2.0 assay (Siemens), and Real-Time HCV genotype II (Abbott)—compared to NS5B sequencing. We studied 327 clinical samples that carried representative HCV genotypes of the most frequent geno/subtypes in Spain. After commercial genotyping, the sequencing of a 367 bp fragment in the NS5B gene was used to assign genotypes. Major discrepancies were defined, e.g. differences in the assigned genotype by one of the three methods and NS5B sequencing, including misclassification of subtypes 1a and 1b. Minor discrepancies were considered when differences at subtype levels, other than 1a and 1b, were observed. The overall discordance with the reference method was 34% for Trugene and 15% for VERSANT HCV2.0. The Abbott assay correctly identified all 1a and 1b subtypes, but did not subtype all the 2, 3, 4 and 5 (34%) genotypes. Major discordances were found in 16% of cases for Trugene HCV, and the majority were 1b- to 1a-related discordances; major discordances were found for VERSANT HCV 2.0 in 6% of cases, which were all but one 1b to 1a cases. These results indicated that the Trugene assay especially, and to a lesser extent, Versant HCV 2.0, can fail to differentiate HCV subtypes 1a and 1b, and lead to critical errors in clinical practice for correctly using directly acting antiviral agents.

## Introduction

Hepatitis C virus (HCV) is a leading cause of death and morbidity that is distributed worldwide. The most recent calculations have estimated an overall world prevalence of 2.8%, and more than 185 million persons are infected in the world [1]. Chronic HCV infection is associated with liver cirrhosis, hepatocellular cancer, liver failure, and even death [2].

Effective chronic hepatitis C treatment has been one of the most important achievements in public health in recent years. This decade is witnessing the introduction of the clinical use of combined direct antiviral agents (DAAs) therapy, with high rates of sustained viral response (SVR) for almost all genotypes, and different strategies for diverse populations (cirrhotic, transplantation, etc.) [3]. Although treatment has proven cost-effective in most settings [4], there is still room for making DAAs affordable for health systems. When this gap is bridged, HCV treatment across the globe may become available for the first time and could open a gateway to cure and eradicate hepatitis C [5].

HCV exhibits a high degree of genetic diversity. HCV strains are classified into seven genotypes, named 1 to 7, with differences of 30–35% of nucleotide sites, and also into 67 confirmed, and some provisional, subtypes with intra-subtype differences of <15% of nucleotide sites [6]. For the time being, the DAA regimen, treatment duration and the need for adjuvant ribavirin partly remain dependent on HCV genotype and subtype [7–9]. Commercial HCV genotyping assays are currently based on different strategies (DNA sequencing, Reverse Hybridization, Real-Time PCR) and distinct HCV genomic targets are used (5’-UTR, Core, NS5B), but there is no commercial assay available that interrogates the NS5B region with DNA sequencing followed by phylogenetic analyses, which is considered the reference method [10]. Several HCV genotype misclassification cases have been recently reported, and have had a negative impact on treatment selection, and consequently on treatment response [11, 12]. In order to achieve the highest degree of treatment response, accurate HCV genotype estimations are mandatory for selecting treatment regimen, and to decide its duration and whether it is necessary to use ribavirin.

In the Spanish Group for Viral Hepatitis Study (GEHEP) of the Spanish Society of Microbiology and Infectious Diseases (SEIMC), we aimed to evaluate the correct assignment of HCV genotypes by three commercial methods -Trugene HCV genotyping kit (Siemens), VERSANT HCV Genotype 2.0 assay (Siemens), and Real-Time HCV genotype II (Abbott)- compared to NS5B sanger DNA sequencing.

## Patients and Methods

GEHEP-007 was an ambispective multicentre study that included 327 clinical plasma samples collected during the 2007–2015 period, with a median (IQR) viral load (Log IU/ml) of 6.27 (5.89–6.68). Samples were representative of the most frequent geno/subtypes that circulate in Spain. The study was conducted in four certified laboratories in Spain. After testing was done by the commercial assay in use in three laboratories [135 samples tested by the Trugene HCV genotyping kit (Siemens), 92 with the VERSANT HCV Genotype 2.0 assay (Siemens), and 100 samples by Real-Time HCV genotype II (Abbott), following the manufacturer’s instructions for them all], an internal fragment of 367 bp in the NS5B gene was amplified and sequenced according to a unique protocol. The Ethics Committee of the San Cecilio Hospital approved the study, and no consent information was required as patient information was anonymised and de-identified prior to analyses.

To ascertain the best NS5B sequencing protocol performance in each laboratory, a proficiency panel was prepared by one of the participating sites and was distributed to the rest. The proficiency panel consisted in 10 samples previously characterised at the Universidad de Navarra by sequencing different genome targets. The specimens that belonged to genotype 1 had been characterised by sequencing Core, NS5A and NS5B regions. Core and NS5B sequencing was used to characterise the remaining genotypes. Laboratories could not enter the study unless agreement with the blinded panel was obtained. A 1 ml aliquot of a 1/5 dilution of the original sample was frozen at -80°c until shipment on dry ice to the other three participating sites. The genotypes and viral load (Log IU/ml) of the samples provided for testing were as follows: 1a_clade I (6.62), 1a_clade II (6.45), 1a_Interclade (5.56), 1b (6.21), 1b (6.59), 2a (5.65), 3a (5.69), 4a (5.33), 4d (5.81), and 5a (5.43).

After RNA extraction by the Magnapure Compact System (Roche Diagnostics, United Kingdom), a fragment of 367 bp encompassing codons 219–342 of the NS5B region was reverse-transcribed and amplified following the modification of a previously published protocol [13] as follows: the first-round RT-PCR reaction was performed with 10 μl of RNA in a final volume of 50 μl of a PCR mixture that contained 1.0 pmol of outer sense (positions 7904 to 7922 H77 based: 5′-TGG GGT TCT CGT ATG ATA CCC-3′) and outer antisense primer (8295 to 8275; 5′-CCT GGT CAT AGC CTC CGT GAA-3′) at 45°C for 45 minutes, followed by 95°C for 15 minutes and 45 cycles of denaturation at 95°C for 30 seconds, annealing at 56°C for 30 seconds, extension at 68°C for 1 minute, and a final elongation step at 68°C for 10 minutes. One microlitre of the first PCR product was subjected to nested PCR with an inner pair of sense (7916 to 7935 5′-GAT ACC CGC TGC TTT GAC TC-3′) and antisense primers (8284 to 8266 5′- CCT CCG TGA ARR CTC KYA G-3′) under the following amplification conditions: denaturation at 95°C for 10 minutes and 30 cycles of denaturation at 94°C for 20 seconds, annealing at 58°C for 30 seconds, extension at 72°C for 1 minute, and a final elongation step at 72°C for 10 minutes.

DNA sequencing was performed using the same primers as for the nested PCR by three different platforms: the Open Gene Sequencing Platform at the San Cecilio Hospital for the Trugene comparison; Applied Biosystems at the Ramon y Cajal Hospital for the Abbott Comparison; Beckman CEQ 8000 at the Complejo Hospitalario Santiago de Compostela for the Versant comparison. After DNA sequencing, HCV genotypes were assigned using geno2pheno_HCV_ (hbv.bioinf.mpi-inf.mpg.de/), MoleBlast (http://hcv.lanl.gov/content/sequence/BASIC_BLAST/basic_blast.html) and phylogenetic trees were reconstructed by maximum likelihood (ML) with PhyML 3.0 using the general time reversible plus proportion of invariable sites, plus gamma distribution and a BIONJ starting tree.

For the data analysis, discordances were classified as *major*, defined as the differences in the assigned genotype by a commercial method and NS5B sequencing (including genotypes 1a and 1b misclassification), and as *minor*, considered when differences at the subtype level were observed.

## Results

The distribution of genotypes across different tests and the three comparative participating centres is shown in Table 1. The majority of samples had been previously screened as genotype 1 (n = 214; 65%), followed by genotype 3 (n = 63; 19%) and genotype 4 (n = 34; 10%). For genotype 1, subtype 1a was pre-screened by commercial tests in 122 samples (57%), subtype 1b in 84 samples (39%), and it was not possible to determine the subtype in 8 samples.

**Table 1:** Pre-screening of the 327 samples included in the study by three commercial assays.

Genotype; [n, (%)]	Subtype	Trugene	VERSANT HCV 2.0	Abbott RT	Total
**1, [**214 (65%)]	1a	33	41	48	122
	1b	47	19	18	84
	Unassigned	6	2	-	8
**2, [**13 (4%)]	2a	1	2	-	3
	2c	1	2	-	3
	Unassigned	-	-	7	7
**3, [**63 (19%)]	3a	23	16	-	39
	3d	1	-	-	1
	Unassigned	2	2	19	23
**4, [**34 (10%)]	4a	8	1	-	9
	4c	12	1	-	13
	Unassigned	-	6	6	12
**5, [**3 (0.9%)]	5a	1	-	-	1
	Unassigned	-	-	2	2
**Total**		135	92	100	327

All three methods used to interpret the NS5B sequence (geno2pheno, Blast, and Phy) gave concordant results in all cases. Regarding the reference method, overall discordance, calculated as both major and minor discordances, was 34% for Trugene, and 15% for VERSANT HCV2.0. The Abbott assay correctly identified all 1a and 1b subtypes, and genotypes 2, 3, 4 and 5, but was unable to discriminate the subtype for the latter, which represented 34% of the cases. Major discordances were found in 16% of the cases for Trugene HCV, and the majority of the discordant cases were due to the commercial assay misclassifying genotype 1b (n = 14; 13 cases reclassified as 1a by NS5B DNA sequencing, and 1 case reclassified as 3a); 5 cases were misclassified as 1a by Trugene, and were further reclassified as 1b by NS5B DNA sequencing. We found major discordances for VERSANT HCV 2.0 in 6% of cases, where all except one case (n = 5) were misclassifications of genotype 1b, which were further reclassified as 1a by the reference method. The other recorded misclassified case was genotype 1, which was further reclassified as 4d by NS5B DNA sequencing. These results are summarized in Table 2.

**Table 2:** Major discordances (differences in the assigned genotype by a commercial method and NS5B sequencing, including the misclassification of subtypes 1a and 1b) from the three assayed commercial tests compared to NS5B DNA sequencing.

Commercial assay result	NS5B DNA Sequencing	Trugene [n (%)]	VERSANT [n (%)]	ABBOTT [n (%)]
**1b**	**1a**	13 (10%)	5 (5%)	-
**1a**	**1b**	5 (4%)	-	-
**1b**	**3a**	1 (0.7%)	-	-
**3a**	**1b**	1 (0.7%)	-	-
**1**	**4d**	-	1 (1%)	-
**4c**	**1b**	1 (0.7%)	-	-
**TOTAL**		**21 (16%)**	**6 (6%)**	**0**

Minor discordances were found for Trugene in 24 samples (18%), most of which were subtype misclassifications of genotype 4 (n = 13), which were further reclassified by NS5B sequencing into different subtypes. However, some cases of genotype 3 (n = 3) and genotype 2 (n = 2) were also reclassified. It is noteworthy that six genotype 1 samples that could not be subtyped by the commercial test were reclassified as subtype 1a (n = 2), and as subtype 1b (n = 4) by the reference method. Minor discordances were recorded in 6 (6%) samples for VERSANT HCV 2.0; it is interesting to note that for this test, all the discordant cases lacked a subtype assignment of genotype 4 (n = 5), genotype 3 (n = 2), and genotype 1 (n = 2; both subtype 1a) with the reference method. With NS5B sequencing, genotypes 2, 3, 4 and 5 (34%) were classified by the Abbott HCV genotype Real-Time II assay as subtypes 2a (n = 2), 2b (n = 2), 2c (n = 3), 3a (n = 19), 4a (n = 2), 4d (n = 4) and 5a (n = 2). The results for the minor discordances are found in Table 3.

**Table 3:** Minor discordances (differences at the subtype level) from the three assayed commercial tests compared to NS5B DNA sequencing.

Commercial assay result	NS5B DNA Sequencing	Trugene [n (%)]	VERSANT [n (%)]	ABBOTT [n (%)]
**1**	**1a**	2 (1%)	2 (2%)	-
**1**	**1b**	4 (3%)	-	-
**2**	**2a/b/c/i**	2 (1%)	-	7 (7%)
**3**	**3a**	3 (2%)	2 (2%)	19 (19%)
**4**	**4a/c/d**	13 (10%)	5 (5%)	6 (6%)
**5**	**5a**	-	-	2 (2%)
**TOTAL**		**24 (18%)**	**9 (9%)**	**34 (34%)**

## Discussion

Hepatitis C Virus genotyping is a key component of proper clinical hepatitis C management [8]. Genotyping, which includes at least subtyping that differentiates 1a and 1b subtypes, needs to be performed before starting antiviral therapy because choice of direct antiviral agents, the need to add ribavirin and treatment duration are directly influenced by HCV genotype [8, 9, 14, 15]. Treatment success and patient cure rates greatly depend on correct HCV genotype identifications. Genotyping may also provide insights into molecular epidemiology studies to know the HCV distribution across the world [16]. Finally, genotyping is also important for interpreting resistance-associated variants [17] as some changes may be common polymorphisms, depending on the specific geno/subtype that infects a patient.

In this study we investigated the accuracy of three commercial assays used for HCV genotyping by sequencing the NS5B region of HCV as the reference method. We initially set up a quality control for three certified laboratories in Spain. After qualification, each laboratory then used a unique NS5B sequencing protocol to re-analyse the clinical samples that were previously tested by the commercial assay used for routine testing at their site. All three labs passed the proficiency panel, although two samples had to be retested at one of the sites. Then 327 routinely screened samples (135 with the Trugene assay, 100 with the Abbott Real-Time assay and 92 with the VERSANT HCV 2.0 assay) were compared to NS5B DNA sequencing.

The HCV genotypic distribution in the sample we used is highly representative of HCV genotypic prevalence in Spain and elsewhere in Europe. According to the May 2015 “Plan Nacional Contra la Hepatitis C” (Spanish Plan against Hepatitis C) [14], genotype 1 is the most prevalent in Spain and represents 69% of the population, followed by genotype 3 which infects 20% of the Spanish population, and lastly by genotype 4, currently detected in 8% of the population. In our study, 65%, 19% and 10% of the samples were HCV genotype 1, 3 and 4 respectively, which is also representative of a recent Spanish genotypic survey [18]. In Europe and the US, several studies [19, 20] have reported a similar distribution of HCV genotypes.

Almost one third of the results obtained by the Trugene assay were discordant with those obtained by NS5B sequencing. Although it is a sequencing method, this commercial assay interrogates only the 5’-UTR region for HCV genotyping. This region is more conserved than NS5B, and although amplification rates are expected to be higher, its potential for genotype (and subtype) discrimination is lower. Indeed in our assay comparison, Trugene failed to correctly classify thirteen 1b subtypes, which were reclassified as 1a subtypes, and five 1a subtypes were reclassified as 1b. In the present-day, correct genotype 1 subtyping is critical to extend treatment duration to 24 weeks, for the addition of ribavirin [7–9, 17, 21, 22], and to also decide on investigating RAVs to retreat patients with prior failure to an NS3- or NS5A DAA-containing regime [9]. Some other studies have also recently reported a frequent number of mistakes using 5’UTR-based assays, and the need to target and/or add other regions for HCV genotyping [23–24].

The Versant HCV genotype 2.0 assay is based on probes which, in addition to the 5’UTR region, target the HCV Core region. With this strategy, genotype/subtype misclassification significantly reduces compared to the Trugene assay. However compared to NS5B sequencing, a number of misclassifications (up to 6%) were still recorded, mainly due to erroneous subtype 1b calls, which were reclassified as 1a. Other researchers have also reported HCV genotype 1 misclassifications when the Versant HCV genotype 2.0 assay was used: Guelfo et al described an 11% misclassification rate obtained by Versant HCV 2.0, which were once again due to 1b subtypes being reclassified following an entire core region as 1a subtypes [25]. Although other researchers have reported lower numbers in misclassifications by Versant HCV 2.0, the main misclassification of the line probe assay (LIPA), as in our study, was for 1b subtypes, which were reclassified into 1a [23]. As in the study of Larrat S et al [26], but unlike those of Avo et al [27], and Quer et al [28], genotype 1 indeterminate calls by the Versant HCV 2.0 test were not frequent in our study. Current treatment guidelines [9] consider treating all 1 subtype indeterminate results as subtype 1a because this is currently “more difficult to treat” than 1b. However, the correct classification of non subtypeable genotype 1 samples would help save treatment options for these patients. Another factor that may lead to misclassification is the viral recombination possibility; Hedskog C, et al [29] reported 12 cases of recombinants, which were identified as different subtypes of genotype 2 by Versant HCV 2.0, were reclassified as subtypes 1a or 1b in NS5B, but were finally classified as recombinants of subtypes 2 and 1 by full genome sequencing.

The Abbott Real-Time HCV Genotype II is based on probes that target both the 5’UTR and NS5B regions. Although we recorded no major discordances for the Abbott assay, Gonzalez V et al [30] were unable to assign any subtype in 29 of 533 (5.4%) cases, Chevaliez et al. had the same trouble in 6.1% of 495 HCV-1 specimens [31], Benedet M et al faced the same problem in 9–10% of the 1052 cases they examined [32], and Ceccherini Silberstein et al reported the same problem in 4% of their 343 patients [33]. Quer et al. also reported major discordances compared to the ultradeep sequencing and population sequencing of the NS5B region [28]. We did not find any genotype misclassification for the non 1 genotype samples, which were investigated by the Abbott assay with probes that targeted the 5’UTR. As only genotype 1 was investigated in this assay with the probes that targeted NS5B, a small sample number for the non 1 genotypes could explain why we found no discordances for these genotypes.

Our study has its limitations. Firstly, not all the samples were tested by the three commercial assays that we compared; we attempted to overcome this limitation using a unique amplification and sequencing protocol, and by accrediting the three laboratories with the proficiency panel distribution. Secondly, the sample number for the non 1 genotypes was low and did not have sufficient discriminative power to draw conclusions for these genotypes, especially for the Abbott assay. Thirdly, only a small portion of the entire NS5B gene was utilised for genotype determination, and we did not perform full-genome sequencing. Hence although recombination is a rare event in HCV, it could be responsible for some of the discordances that we reported. Allthough no mixed infections were detected among our samples, they could also explain some misclassifications. Finally, the Trugene assay is no longer available commercially, but we believe that our results may be valuable for those patients who were genotyped in the past by this test and are now candidates for treatment with DAAs.

Despite these limitations, and considering that commercial assays meet diagnostic laboratory expectations in turn-around time and sensitivity terms, our results indicated that the Trugene assay, especially, and to a lesser extent, Versant HCV 2.0, could fail to differentiate HCV subtypes 1a and 1b, which would lead to critical errors in clinical practice for correctly using directly acting antiviral agents. In spite of the high cost of HCV antiviral therapy, implementing reference tests for HCV genotyping in clinical microbiology laboratories, such as NS5B Sanger sequencing, which has a relatively low cost compared to treatment, may help improve commercial assays.
